# Intelligent Recognition and Analysis of Negative Emotions of Undergraduates Under COVID-19

**DOI:** 10.3389/fpubh.2022.913255

**Published:** 2022-05-18

**Authors:** Weifeng Zhang

**Affiliations:** School of Educational Science, Xinxiang University, Xinxiang, China

**Keywords:** COVID-19, undergraduates, negative emotion, intelligent recognition, data analysis

## Abstract

**Background:**

The outbreak and spread of COVID-19 has brought a tremendous impact on undergraduates' study and life, and also caused anxiety, depression, fear and loneliness among undergraduates. If these individual negative emotions are not timely guided and treated, it is easy to cause the amplification of social negative emotions, resulting in individual and collective irrational behavior, and ultimately destroy social stability and trust foundation. Therefore, how to strengthen the analysis and guidance of negative emotions of undergraduates has become an important issue to be urgently solved in the training of undergraduates.

**Method:**

This paper presents a weight and structure double-determination method. Based on this method, a Radial Basis Function Neural Networks (RBFNN) classifier is constructed for recognizing negative emotions of undergraduates. After classifying the input psychological crisis intervention scale samples by the RBFNN classifier, recognition of negative emotions for undergraduates are divided into normal, mild depression, moderate depression and severe depression.

**Experiments:**

Afterwards, we analyze negative emotions of undergraduates and give some psychological adjustment strategies. In addition, the experiment results demonstrate that the proposed method has a good performance in terms of classification accuracy, classification time and recognition rate of negative emotions among undergraduates.

## Introduction

COVID-19 is having a great impact on the study and life of undergraduates all over the world. Facing the major pandemic, undergraduates should pay attention to not only their physical health, but also their emotional and psychological health (1, 2). Since the outbreak at the end of 2019, COVID-19 has spread rapidly around the world in a short period of time, and the pandemic prevention situation is very serious. The pandemic crisis is not only affecting people's physical health, and it is also inevitable to have an emotional impact. On the one hand, when undergraduates browse Weibo and other self-media, they will face the explosive circulation of various pandemic-related information, which will have a psychological impact. On the other hand, undergraduates need to adapt to the comprehensive online teaching state and complete various learning tasks arranged by the school. In such a situation, undergraduates will be in a stressful emotion for a long time, loneliness, anxiety, depression, fear and other negative emotional states will appear. If not paid attention to and adjusted in time, more problems and obstacles may arise (3). Therefore, both undergraduates themselves and universities should pay close attention to the emotional health of undergraduates while paying attention to the pandemic and their studies, so as to make preparations for returning to normal learning and life.

Currently, undergraduates have strong expressions, but their psychological quality is poor and they are easy to fall into emotional troubles. Emotions of undergraduates are characterized by stability and volatility, complexity and hierarchy, impulsiveness and rationality, externality and implicitness (4, 5). Especially in the COVID-19 pandemic, a series of negative emotions such as anxiety, inferiority complex, resentment and jealousy are sometimes aroused. Negative emotions affect the physical and mental health of undergraduates, which makes it difficult for them to create harmonious relationships between individuals and others, stifle their creativity and lead to destructive or aggressive behaviors (6–8). Appropriate application of emotional venting, emotional transfer and emotional sublimation can effectively correct the negative emotions of undergraduates and promote their mental health.

Negative emotions will not only harm the physical and mental health of undergraduates, and sometimes even extreme behavior, leading to personality splitting among undergraduates. An important characteristic of personality splitting is the separation of external self and internal self (9). One is internal inferiority and external arrogance, concealing inferiority with arrogance; Secondly, the internal pursuit of external denigration, all their external performances are false, and do not reflect the real thoughts of the undergraduates themselves, which is out of the opposite value of the external performance of the desire; Thirdly, the internal hatred and external humility, which is mainly manifested as appreciation or praise. It is also a distortion or alienation of praise, and the undergraduates' external humility cannot give up their internal hatred.

Harmonious interpersonal relationship is conducive to the growth and progress of undergraduates, but undergraduates with strong negative emotions will put their interpersonal relationship in a state of tension, and lack of positive interaction with teachers and classmates. Especially in the tense state of COVID-19, it is impossible to create a harmonious relationship between themselves and others. Taking jealousy as an example, the most important manifestation of jealousy is self-prominence. One is to directly belittle others, to belittle their ability, quality and wisdom. The other is indirect demeaning, through praise, flattery and other forms to ridicule others, it can be seen that jealousy will inevitably weaken the importance of others, leading to the cold relationship between the two sides (10, 11). Especially with COVID-19, communication with classmates and teachers is already rare, which is more likely to lead to the extreme development of problems.

Negative emotions will demote the creativity of undergraduates. Taking learning as an example, when some poor students are incapable of achieving excellent results, they may be jealous of excellent students, which easily leads to their denigration and negation to excellent students, abandoning their pursuit for high value, making themselves mediocre and demoting their creativity (12). Under COVID-19, work is very important for undergraduates. If they do not achieve good results, finding a job is a luxury for them. At the same time, the negative emotions of undergraduates will demote the creativity of others. The undergraduates with negative emotions cannot tolerate the existence of independent personality and excellent quality. If they are jealous of excellent undergraduates, they will defame, attack, distort and depreciate them (13). When excellent undergraduates cannot resist defamation, they will choose to conform to the public and make themselves mediocre. Negative emotions not only demote the creativity of undergraduates, but also lead to destructive and aggressive anti-moral behaviors. The generation of negative emotions of undergraduates is fundamentally the result of unsatisfactory individual needs, such as security, belonging, self-esteem and love. When these needs are lacking or deprived, undergraduates will behave destructively and aggressively. The destructive or aggressive behavior of undergraduates is not only verbal but also behavioral. Undergraduates with strong negative emotions can be attacked and destroyed completely by unrelated people or things, even make harmful acts against the body of teachers and students, or even take extreme measures (14).

Emotion is a general term for a series of subjective cognitive experiences, which is also a psychological and physiological state resulting from the combination of multiple feelings, thoughts and behaviors. People's emotions are often reflected in their gestures, facial expressions, and speech. As the development of computer technology, people hope to endow computers with emotional ability, so that computers can interact with people emotionally. Making computers to accurately recognize human emotions is a key technology for achieving this vision. Common emotion recognition methods are based on facial expression, text content, physiological signals and speech (15). The objective of this paper is to propose a negative emotion recognition method based on psychological crisis intervention scale for undergraduates. Emotion recognition research generally includes six steps: emotion induction, signal acquisition, data preprocessing, feature extraction, feature dimension reduction, emotion learning and classification. However, this paper mainly studies the classifier to achieve negative emotion recognition and analyzes the negative emotions of undergraduates under COVID-19 through psychological crisis intervention scale.

Accordingly, the main contributions of this paper are summarized as follows. (i) A weight and structure double-determination method is proposed to solve the problems of hidden neuron center, variance of Radial Basis Function Neural Networks (RBFNN) and difficulty in determining network weight and topology. (ii) We analyze the negative emotion of undergraduates by using the results of RBFNN classifier.

The rest paper is structured as follows. Section Related Work reviews the related work. In Section Negative Emotions Recognition Method, RBFNN classifier is used for recognizing negative emotions of undergraduates. The experiment and results analysis are shown in Section Experiments and Analysis, and Section Conclusions concludes this paper.

## Related Work

### Emotion Recognition

Emotion is an important part of people's life. Accurate emotion recognition has great significance and application value in the fields of interpersonal communication, diagnosis and treatment of emotional disorders and other fields. Emotion recognition can be divided into non-physiological signals (such as facial expression, speech, behavior) and physiological signals (such as electrocardiogram, electroencephalogram) from the perspective of signal source. Khattak et al. (16) proposed an efficient deep learning technology, which used convolution neural networks to classify emotions from facial images and effectively detect age and gender from facial expressions. Zhang and Zhang (17) proposed an automatic encoder with emotion embedding to extract deep emotional features. Panahi et al. (18) studied the effectiveness of fractional Fourier transform as a new feature extraction method in improving the accuracy of emotion recognition in physiological signals. Liu et al. (19) proposed a hybrid feature extraction method based on empirical mode decomposition domain and combined the optimal feature selection method for emotion recognition of electroencephalogram signals.

### Neural Networks Classifier

Classification has always been a core issue in the fields of machine learning, pattern recognition and data mining. Feng et al. (20) proposed an Enhanced Swarm Intelligent Clustering (ESIC) method to select hidden layer neurons and then train cosine RBFNN based on gradient descent learning process. Cruz et al. (21) proposed a bee-inspired data clustering approach to design RBFNN classifier (BeeRBF). Wen et al. (22) presented an incremental learning algorithm for the hybrid RBF-back propagation (ILRBF-BP) neural network classifier. Chan et al. (23) introduced a multi-classifier method to improve the performance of neural network designed in series and reduce the pressure of the final classifier.

### Emotion Analysis of Undergraduates Under COVID-19

At present, academic researches on negative emotions of undergraduates under the COVID-19 pandemic are mainly carried out from the perspectives of ideological and political education, psychological assistance and intervention. They really focus on emotions themselves, and there are few researches on the causes, formation mechanism, forms of expression and transmission characteristics of negative emotions of undergraduates. Zhu et al. (24) studied the mental health and emotion regulation experience of Chinese nursing undergraduates during covid-19, which was helpful for direct psychological intervention during the pandemic. Deng et al. (25) showed that the greater the COVID-19 related stress, the higher the sexual compulsivity, and the longer the duration of undergraduates' anxiety. Rahimi and Vallerand (26) studied the role of academic enthusiasm and emotion in procrastination. Dantas et al. (27) studied how the COVID-19 pandemic affected undergraduates' emotion and the learning status of Brazilian undergraduates during the suspension of classes in 2020. Moeller et al. (28) discussed whether some risk factors and protective factors could predict undergraduates' emotions and individual internal changes caused by the outbreak of the pandemic.

Given the above, there are few articles recognizing and analyzing negative emotions of undergraduates under COVID-19. Therefore, this paper conducts research on this issue.

## Negative Emotions Recognition Method

Due to the advantages of simple network structure, good approximation ability and generalization ability, RBFNN is widely used in data approximation, pattern recognition and other fields. This paper uses RBFNN classifier for recognizing negative emotions of undergraduates. RBFNN is a single hidden layer forward neural networks model (29).

There are four main factors that determine the performance of RBFNN: RBF center, variance, connection weight between hidden layer and output layer, and number of hidden layer neurons. If the center or variance of hidden neurons are not selected correctly, the learning error of the network will be too large and the performance of the network will be reduced. The calculation of connection weight between hidden layer and output layer of neural networks also has a great impact on the performance of the network. The number of hidden layer neurons can also affect the performance of the network. If the number is too small, the learning and approximation ability of the network will be insufficient. While if the number is too large, the response speed of the neural network will be reduced, and even the over-fitting phenomenon will occur (30). Therefore, RBFNN learning and training process mainly revolves around these four factors. In the process of negative emotion recognition based on psychological crisis intervention scale in psychological census, this paper proposes a weight and structure double-determination method to solve the problems of hidden neuron center, variance of RBFNN and difficulty in determining network weight and topology.

### Direct-Weight-Determination Method

**Figure 1:**
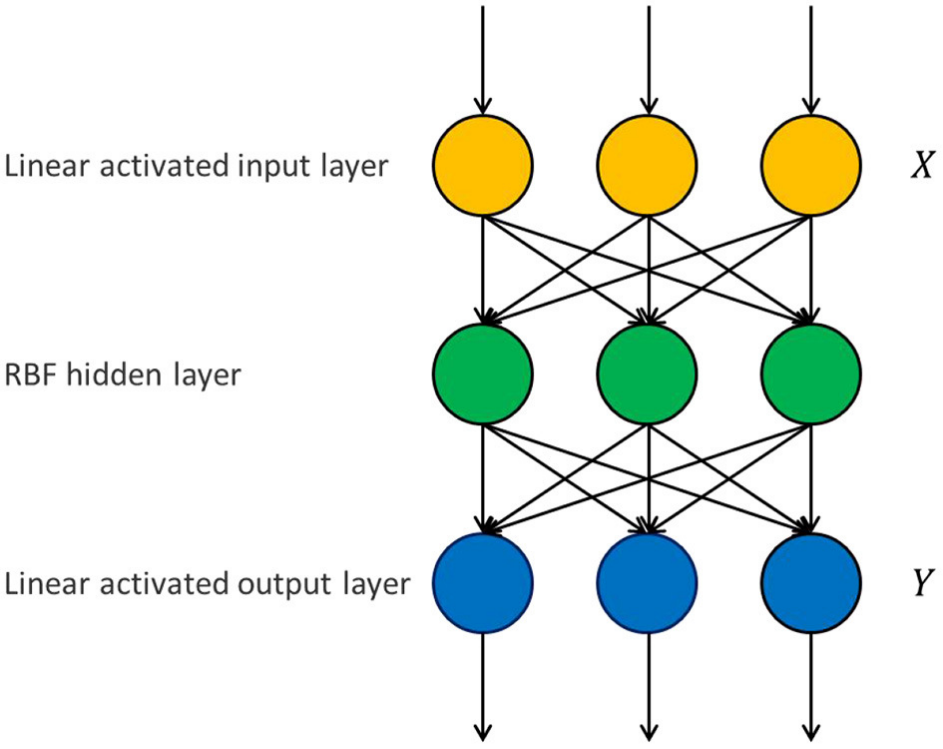
RBFNN classifier model.

RBFNN classifier consists of input layer, hidden layer and output layer, and its architecture is shown in Figure 1. The input layer obtains input data and transmits it to the hidden layer. The activation function used by the hidden layer neurons is the radial basis function, and the output result of the output layer neurons is the weighted sum of the output of the hidden layer neurons.

For the connection weights between hidden layer and output layer of RBFNN, the traditional method is based on the idea of negative gradient and obtained by iterative learning. However, using iterative method to train neural network has the problem of time-consuming iterative process (31). In this paper, a direct-weight-determination method based on pseudo-inverse is used to obtain the optimal connection weight between neurons in hidden layer and output layer.

Let the number of training samples be *J*, then the input matrix of the training sample set is *X* = (*x*_1_, *x*_2_, ⋯ , *x*_*J*_), and the expected output matrix of the training sample set is *A* = (*a*_1_, *a*_2_, ⋯ , *a*_*J*_).

The optimal connection weight between hidden layer and output layer of the three-layer forward neural networks shown in Figure 1 can be directly determined as follows.


(1) $$\begin{array}{l} w={\left(V^{T}V\right)}^{-1}V^{T}A^{T} \end{array}$$

where (*V*^*T*^*V*)^−1^ *V*^*T*^ is the pseudo-inverse of matrix *V*, denoted as V^*^, so Equation (1) can also be defined as follows.


(2) $$\begin{array}{l} w=V^{*}A^{T} \end{array}$$

The activation matrix *V* is defined as follows.


(3) $$\begin{array}{l} V=\left[\begin{array}{cccc} p_{11} & p_{12} & \cdots & p_{1N}\\ p_{21} & p_{22} & \cdots & p_{2N}\\ \vdots & \vdots & \ddots & \vdots \\ p_{J1} & p_{J2} & \cdots & p_{JN} \end{array}\right] \end{array}$$

where $p_{jn}=\exp\left(-\frac{{\left\|x-\mu_{n}\right\|}^{2}}{2\sigma_{n}^{2}}\right)=exp\left(-\frac{1}{2\sigma_{n}^{2}}\sum_{k=1}^{K}{\left(x_{jn}-\mu_{jn}\right)}^{2}\right)$ represents the response value of the *n*th hidden layer neuron activated by sample *j*.

Without loss of generality, the average output error of the network is defined as follows.


(4) $$\begin{array}{l} E=\frac{{\left\|A-Y\right\|}_{F}^{2}}{S\times Q} \end{array}$$

where *A* represents the expected output matrix of the network, *Y* represents the actual output matrix of the network, *S* represents the number of samples, *Q* represents the number of rows of the expected output matrix *Y*, and ${\left\|A-Y\right\|}_{F}^{2}$ represents the Frobenius norm of the matrix.

### Double-Determination Method

When determining the weight and structure of the network by using the weight and structure double-determination method, we divide the psychological crisis intervention scale data sample used in this paper into three subsets: training dataset, validation dataset and test dataset.

To clearly describe the weight and structure double-determination method clearly, we give the basic steps for the algorithm. At first, the variables involved are described as follows.

*EC*_min_: minimum network average error correction.

*EC*_*cur*_: current network average error correction.

*N*_*m*_: current number of hidden layer neurons.

*N*_*h*_: optimal number of hidden layer neurons.

*N*_*f*_: number of forward searching neurons.

*N*_*c*_: counter with initial value 0.

The weight and structure double-determination method includes seven steps.

Step 1: Parameters initialization. *EC*_min_ and *EC*_*cur*_ are set to 1000, *N*_*m*_ and *N*_*h*_ are set to 2, the center of RBF is selected as the first training sample input *x*_1_ and the last training sample input *x*_*J*_, variance $\sigma_{1}=\sigma_{2}=\frac{\left\|x_{1}-x_{J}\right\|}{2}$, and *N*_*f*_ is set to the smallest integer >10% of the number of training samples.

Step 2: Whether condition *N*_*c*_ < *N*_*f*_ is satisfied. If it is, go to Step 3; If not, go to Step 6.

Step 3: According to the training samples, the optimal connection weight *w* between hidden layer and output layer of the RBFNN is calculated by using the direct-weight-determination method.

Step 4: Calculate the network error correction *EC*_*cur*_ by using correction sample. If *EC*_*cur*_ < *EC*_min_, then *EC*_min_ ← *EC*_*cur*_, *N*_*h*_ ← *N*_*m*_, and *N*_*c*_ ← 0. Otherwise, delete the newly added neurons, *N*_*m*_ ← *N*_*m*_ − 1, and *N*_*c*_ ← *N*_*c*_ + 1.

Step 5: If all training sample inputs have been used as neuron centers, go to Step 6. Otherwise, add a new neuron, that is, *N*_*m*_ ← *N*_*m*_ + 1, in which the center is selected uniformly from the input of the training sample and the variance is determined by $\sigma =\sigma_{1}=\sigma_{2}=\cdots =\sigma_{n}=\frac{D_{\max}}{\sqrt{2N_{m}}}$. *D*_max_ represents the maximum distance between centers, that is, the maximum norm distance between centers. Then, return to Step 2.

Step 6: After the training of RBFNN, hidden neuron center, variance, network architecture and connection weight *w* between hidden layer and output layer are determined. The network output *y*_*t*_ corresponding to the input vector *x*_*t*_ of the test sample can be calculated and classified. If *y*_*tr*_ = *max*{*y*_*t*1_, *y*_*t*2_, ⋯ , *y*_*tR*_}, *y*_*tr*_ = 1, otherwise *y*_*tr*_ = 0.

Step 7: Decide the type of the vector after classification. When the processed output vector is *y*_*t*_ = [1, 0, ⋯ , 0], it belongs to the first type. When the processed output vector is *y*_*t*_ = [0, 1, ⋯ , 0], it belongs to the second type, and so on.

## Experiments and Analysis

### Dataset

The dataset used in this paper comes from 2000 psychological test reports of undergraduates of different grades in Xinxiang University, including 569 in freshmen, 506 in sophomores, 446 in juniors and 479 in seniors, which is so-called true. The parameters setting for RBFNN is shown in Table 1. To avoid overfitting, we divide the psychological crisis intervention scale dataset into training sample set, validation sample set and test sample set according to Table 1, among which the number of training samples accounts for 51.30% of the total number of samples, the number of validation samples accounts for 19.65%, and the number of test samples accounts for 29.05%.

**Table 1:** Parameters setting for RBFNN.

**Parameter**	**Setting**
Input size	2,000^*^96
*EC*_min_	1,000
*EC*_*cur*_	1,000
*N*_*m*_	2
*N*_*h*_	2
Number of dataset samples	2,000
Number of training dataset samples	1,026
Number of validation dataset samples	393
Number of test dataset samples	581

Since psychological crisis intervention scale is applied to the recognition of negative emotions of undergraduates in this paper, there is no mature negative emotion-related dataset and emotion label dataset. Therefore, starting from collecting psychological crisis intervention scale and emotion label data of some undergraduates, this paper constructs the dataset related to negative emotions required by this paper. We used the psychological crisis intervention scale of 300 undergraduates as the dataset.

### Recognition of Negative Emotions for Undergraduates

The recognition process of negative emotions of undergraduates by RBFNN classifier based on weight and structure double-determination method is described as follows.

Step 1: Undergraduates should answer 96 questions in the psychological crisis intervention scale within a specified time. The scale is compiled according to the age, psychological characteristics and social status of undergraduates and the characteristics of university management.

Step 2: In the process of data acquisition, because some questions may not be answered by undergraduates, there is a certain proportion of invalid data, or noise data, which needs to be filtered out to ensure the accuracy of training, verification and testing.

Step 3: There are 96 questions in the psychological crisis intervention scale for undergraduates, that is, the dimension of the dataset is 96. Such a large feature dimension will slow down the running speed of the machine learning modeling algorithm and increase the possibility of overfitting, leading to the sparser distribution of data in the entire input space and the more difficult to obtain representative samples for the whole input space. Therefore, this paper adopts Principal Component Analysis (PCA) algorithm (32) to extract features from psychological crisis intervention scale of undergraduates, and selects the most important features as the input of RBFNN. PCA is a linear dimension reduction method that converts multiple variables into a few comprehensive variables, i.e., principal components, by studying the internal structure of correlation matrix or covariance matrix of original variables. These transformed principal components can reflect most of the information of variables in the original 96 psychological crisis intervention scale.

Step 4: RBFNN training. The training set is used for the data samples fitted by the model of negative emotion recognition of undergraduates. It is directly involved in the process of model adjustment, and obviously cannot be used to reflect the real ability of the model, that is, to prevent overfitting. A validation set is a set of samples set aside during network training, which can be used to adjust the hyperparameters of the model and preliminarily evaluate the capability of the model. It is usually used to verify the current model generalization ability during model iterative training, so as to decide whether to stop continuing training. The validation set is involved in the process of manual parameter tuning (hyperparameter) and cannot be used to judge a model, but it determines the number of hidden layer neurons in the neural networks. The test set is used to evaluate the generalization capability of the model.

Step 5: The emotions of undergraduates are taken as the output of the neural network, and the RBFNN is used to learn according to the optimal parameters. The relationship between the recognition characteristics and states of undergraduates' negative emotions is fitted, and the recognition model of undergraduates' negative emotions is constructed.

Step 6: Test samples are used to verify the performance of the negative emotion recognition model of undergraduates, and output the negative emotion recognition results of undergraduates, as shown in Table 2. As can be seen from Table 2, after classifying the input psychological crisis intervention scale samples by the RBFNN classifier with weight and structure double-determination method, there are four categories such as normal, mild depression, moderate depression and severe depression. The corresponding score range and output vector can also be obtained from Table 2. In addition, we can see that a small number of hidden layer neurons can alleviate or even avoid the problem of neural network overfitting and improve the noise resistance of the classifier.

**Table 2:** Negative emotion recognition results for undergraduates.

**Emotions**	**Recent risk score range**	**Output vector**	**Number of hidden layer neurons**	**Proportion / 100%**
Normal	0–4	[1,0,0,0]	3	0.7130
Mild depression	5–13	[0,1,0,0]	8	0.2130
Moderate depression	14–20	[0,0,1,0]	7	0.0462
Severe depression	Above 21	[0,0,0,1]	15	0.0278

### Results Analysis

Undergraduates are still in the mature stage of psychological development. Facing the double pressures of COVID-19 and study, they are more likely to breed negative psychological emotions, and even affect their physical health in serious cases. We classify emotions of undergraduates by RBFNN classifier of weight and structure double-determination Method. Besides normal emotion, we analyze other three negative emotions such as mild depression, moderate depression and severe depression caused by psychological crisis.

#### Mild and Moderate Depression

In addition to medication, the treatment and psychological intervention of mild depression and moderate depression are basically the same, so we analyze mild depression and moderate depression together. The common manifestations of mild and moderate depression among undergraduates in COVID-19 are as follows. (i) Cognitive bias. Take a negative view of people and things and complain about the current situation. (ii) Depression and malaise. (iii) Social withdrawal, showing indifference and disinterest in study and life. Undergraduates are in the stage of active thinking and energetic life, but they are still in the special stage of immature. Sudden changes in living environment can also trigger depression, especially during COVID-19, when students are more likely to become distracted by days or months of isolation. There are many reasons for mild and moderate depression, so corresponding psychological adjustment strategies should be multi-dimensional.

##### Receive Professional Psychological Counseling

Universities should set up formal mental health centers with professional psychological counselors and train part-time psychological counselors, and carry out extensive mental health education. This provides a good environment for the adjustment of undergraduates with mild and moderate depression.

##### Participate in Varieties of Campus Activities

The so-called campus culture is a kind of school culture spirit and atmosphere with the specific environment as the background. Normally, campus culture develops simultaneously with social culture. When individuals are integrated into the group, they are sufficiently influenced by the campus culture and show their talents in rich campus cultural activities, thus gaining sufficient confidence. In this way, undergraduates with mild and moderate depression tend to become optimistic and cheerful. But the remarkable thing is that under COVID-19, we should strengthen our protection measures.

In addition, for undergraduates with mild and moderate depression, timely timing of treatment and active self-regulation are the premise to reduce the harm and stabilize the condition.

##### Exercise

Different forms of exercise can reduce stress, relieve depression, and make undergraduates energetic. As a whole, exercise therapy is safe, effective and simple. Combined with the characteristics of mild and moderate depression and various sports, we introduce four kinds of sports. (i) jogging. Jogging can improve sleep and health. (ii) Table tennis. Table tennis exercises the whole body, making more focused and flexible. (iii) Mountain climbing. Mountaineering is a relatively difficult process. While also seeing the spectacular scenery while overcoming difficulties. For undergraduates with mild and moderate depression, mountaineering is not only a process of physical exercise, but also a process of perseverance, which can improve negative thinking to some extent. (iv) Yoga. One of the benefits of yoga is to help people relax, eliminate physical discomfort with the most natural therapies, and give people a power that comes from the deepest part of their heart.

Appropriate exercises can help to cultivate undergraduates' open-minded and cheerful character and enhance their frustration tolerance and psychological quality. In self-training and competition, the improvement of personal exercise performance can enhance self-confidence, taste the joy of success, make self-state of mind in a pleasant environment, and help to alleviate depression. In the collective exercises training and competition, the tacit understanding cultivated with teammates cannot only win the competition, but also gain friendship, which greatly enhances the self-confidence and self-esteem. The personal charm and growing self-confidence during exercise can make up for the inferiority of undergraduates with mild and moderate depression in other aspects.

##### Social Live

Undergraduates with mild and moderate depression are often socially isolated and tend to lose social support over time. It is believed that good social support is beneficial to physical and mental health, while the existence of bad social relations is harmful to physical and mental health. According to relevant studies, undergraduates' validation and support from others, companion and intimacy, and satisfaction are significantly negatively correlated with Symptom Checklist - 90's (SCL-90's) dimensions of depression, psychosis, and interpersonal sensitivity. Conflict and punishment from significant others are positively correlated with the nine dimensions of SCL-90. Therefore, social support plays an important role in the development of undergraduates' mental health, and the establishment and utilization of good social relationship network should be emphasized in the self-treatment of mild and moderate depression in undergraduates.

##### Piano Music

Piano music therapy is one of the most important parts of music therapy. At present, its development in China still belongs to the stage that needs further practice and research. It is a serious and cautious process to select undergraduates with mild and moderate depression from the library of various periods, types and styles. Piano art can cultivate sentiment and improve undergraduates' artistic appreciation and music accomplishment. The significance of piano music therapy is to relieve and improve the symptoms of mild and moderate depression. As the process of piano music therapy is full of relaxation and entertainment, it is easier to be accepted by college undergraduates.

##### Diet Regulation

For diet of undergraduates with mild and moderate depression, they can have some foods containing amino acids and vitamin B, such as fish, vegetable, eggs, etc. In addition to psychological self-regulation, undergraduates should also develop good living habits at ordinary times and get enough sleep.

##### Do Some Interesting Things

If undergraduates are not successful in their studies, find ways to improve their skills, start with things that they are interested in, or find other opportunities for success, and do activities in a planned way that can gain happiness and confidence, especially on weekends, such as cycling, writing, shopping, etc.

Mild and moderate depression rarely interferes much with normal life, but can become very severe when major events happen. In COVID-19, undergraduates should learn to protect themselves, and we should remember “You cannot do anything about the length of your life, but you can do something about its width and depth” mentioned by H.L. Mencken.

#### Severe Depression

For undergraduates with severe depression, in addition to drug intervention, we will analyze it in psychological intervention. Severe depression is a surgical disease that does great harm to undergraduates' physical and mental health. In addition to drug treatment, active nursing of severe depression is needed to help undergraduates effectively relieve their condition. The psychological state of undergraduates with severe depression is very complex and subtle. Most of the time, the expression of some symptoms is only a small aspect, and more painful experience is often unspeakable. So how should we actively intervene?

(1) Make undergraduates with severe depression as happy as possible, relax appropriately and do something that will make them happy. Happiness in the present can help prevent future depression. Bringing happiness into life is not only one of the basic strategies for having a good mood, but also an effective method for severe depression.

(2) Undergraduates with severe depression should be helped to re-establish their values and goals in life. With clear values and goals, they can realize whether their current study and personal life conform to their own values, thus effectively inhibiting the development of depression, which is of good help for the treatment of severe depression.

(3) Families can help undergraduates build reliable relationships. Clinical studies show that undergraduates with severe depression should talk to their families, classmates and friends, which is conducive to suppress depression. Severe depression patients are very sensitive, and they are likely to have psychological fluctuations in life due to one word, one action and one thing. Therefore, families should pay attention to their words and actions in front of undergraduates so as to avoid psychological irritation to them. As a family of undergraduates with severe depression, psychological or verbal support is not enough, but more important is action support. Recovery from severe depression is not achieved overnight, so families should first maintain maximum patience and show confidence and hope in front of undergraduates. As long as families do not give up and have confidence, it will surely drive the confidence and hope of undergraduates.

As undergraduates with severe depression, due to the change of their personality structure, they should enhance their self-confidence, form a more optimistic and positive way of thinking, gradually change the bad psychological state, and strive to change the negative life cognition, which is conducive to psychological rehabilitation. At the same time, for undergraduates with suicidal tendencies, we should let them realize that everything is possible to solve, relieve their sense of despair caused by severe depression, so as to reduce the possibility of suicide.

#### Strategies for Effectively Dealing With Negative Emotions of Undergraduates

Undergraduates have poor ability to deal with negative emotions. On the one hand, the reason is that undergraduates have been living in a relatively simple world of campus, with narrow contact range, less life experience and relatively slow psychological development. On the other hand, due to the pre-college education model that emphasizes academic performance over emotional intelligence, undergraduates lack methods to deal with negative emotions. Negative emotions of undergraduates mainly come from the following three aspects. (i) Academic pressure. (ii) Influence of home environment. (iii) Complex personal connections.

Negative emotions of undergraduates include frustration, sadness, shame, fear, grief, guilt, depression, despair, envy, doubt and jealousy. Undergraduates are in a period of changeable emotions. It is normal for them to have various negative emotions when they meet setbacks and failures, but they cannot let the negative emotions control themselves. Therefore, they should use appropriate methods to effectively correct their negative emotions so that they can face the reality, accept the reality, maintain a healthy attitude and have a good ability to adapt to the environment. This is not only the guarantee of undergraduates' mental health, but also the necessary condition for undergraduates to improve their personality and self-improvement.

##### Emotion Catharsis

Reasonable venting of negative emotions through certain channels is an effective way to correct negative emotions. Emotional catharsis has direct and indirect two ways. Direct catharsis means expressing one's own emotions to the person or thing causing the negative emotions, while indirect catharsis means expressing one's own emotions to the person or thing other than the negative emotions, or venting excess energy through certain activities so that the mental and spiritual health can be regulated. Therefore, when encountering what are not working, we should take the initiative to find families or friends to exchange ideas, communicate feelings, enhance confidence and overcome negative emotions. People who communicate and interact well are often more likely to be healthy, happy and successful.

##### Emotion Transfer

Emotion transfer is a method of consciously transferring negative emotions to other people or things to create new excitement points for release. When unpleasant things happen, do something that is usually liked, such as listening to music. The music with bright rhythm and smooth melody has the effect of inspiring spirit, while the music with slow rhythm and clear melody has the effect of calming and comforting. Or participate in meaningful sports activities such as running, playing ball and swimming, which not only exercise the body, but also eliminate emotional tension and anxiety. Undergraduates should learn to consciously control and correct their own emotions and overcome the interference of negative emotions with strong will.

##### Emotion Sublimation

Emotional sublimation refers to guiding negative emotions to the direction that is beneficial to people, oneself and society, so as to make them conform to the behavioral norms required by society and the requirements of the progress and development of the times. It is a high-level catharsis of negative emotions.

In the management of colleges, measures should be taken to improve the ability of undergraduates to deal with negative emotions, and at the same time, external helps should be provided for undergraduates to deal with negative emotions. For example, run emotional management course, emphasize the function of physical exercise, guide cultural and recreational activities, strengthen dormitory construction, strengthen catering construction, and strengthen the management capability of counselors.

#### Comparison for Classification

**Figure 2:**
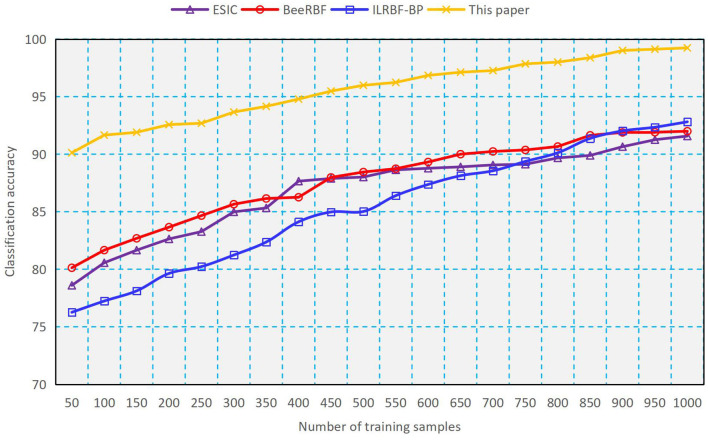
Performance of classification accuracy.

**Figure 3:**
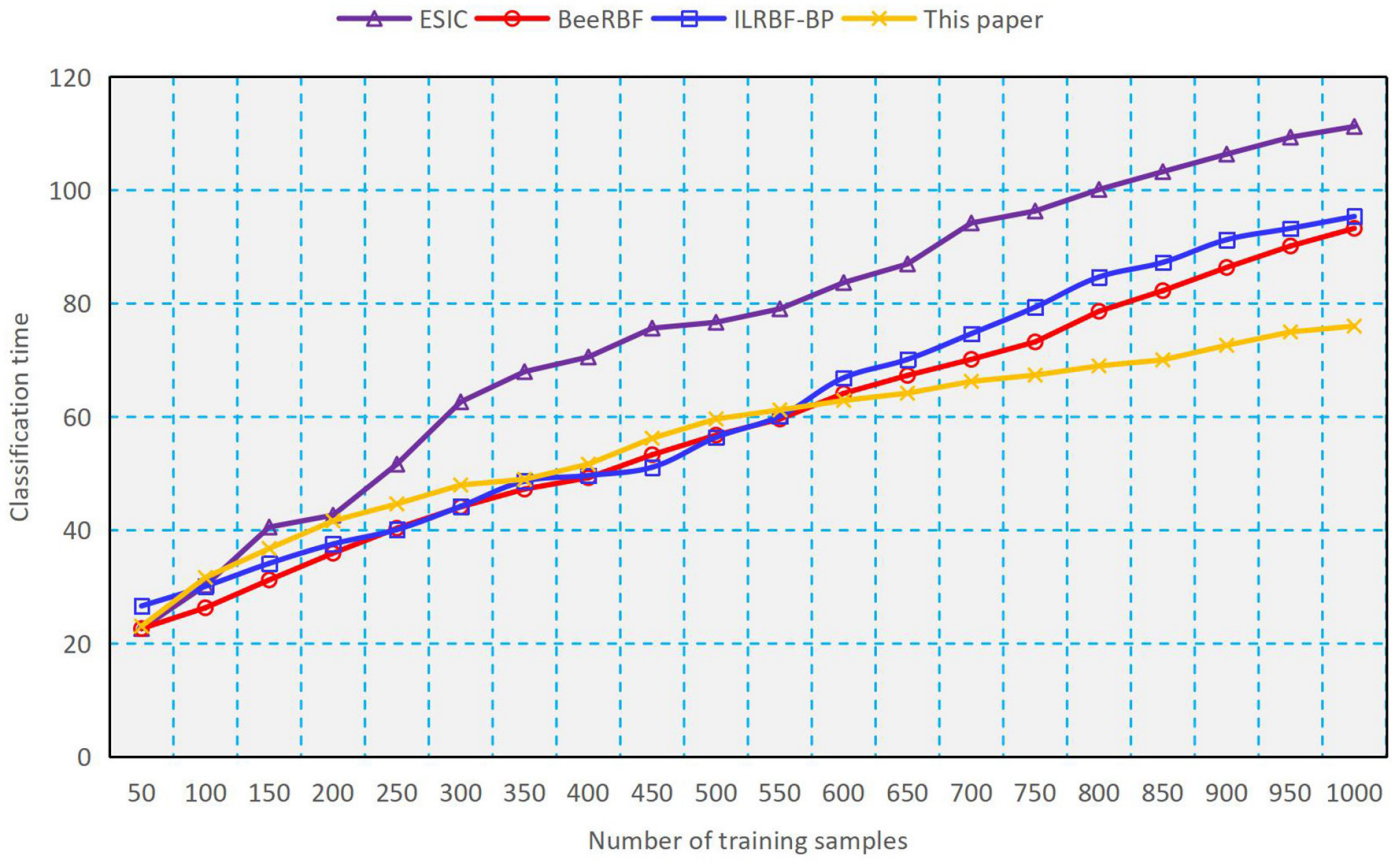
Performance of classification time.

**Figure 4:**
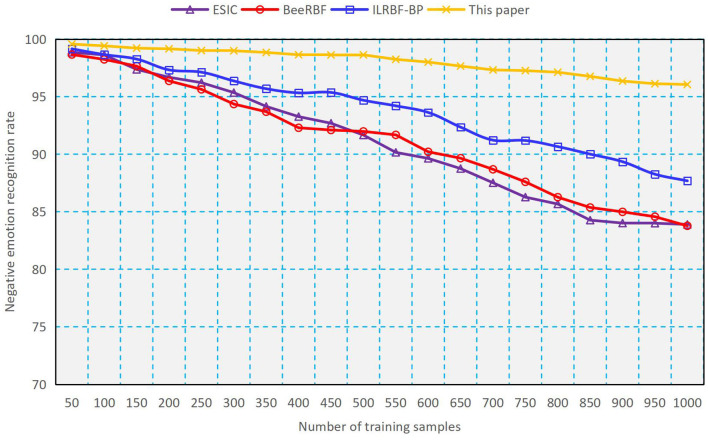
Performance of Negative emotion recognition rate.

As a comparison, we use four methods such as ESIC (20), BeeRBF (21), ILRBF-BP (22) and the proposed double-determination method to train the RBFNN, and the results of classification accuracy and classification time obtained are shown in Figures 2, 3. It can be seen from Figures 2, 3 that with the increasing number of training samples, using the proposed method to train RBFNN has a high classification accuracy and a low classification time, which shows the validity of the weight and structure double-determination method proposed in this paper. In addition, we compare the recognition rate of negative emotions of undergraduates by using different methods. As indicated in Figure 4, the recognition rate of negative emotions of the proposed double-determination method is always higher than 95%.

## Conclusions

This paper recognizes and analyzes the negative emotions of undergraduates under COVID-19, designs and proposes a double-determination method of network weight and structure, which can be used to determine the center, variance and number of hidden layer neurons of RBFNN and the optimal connection weight between hidden layer and output layer. Furthermore, we analyze common of mild and moderate depression among Undergraduates. Meanwhile, for undergraduates with mild and moderate depression, treatment and active self-regulation are the premise to reduce harm and stabilize the condition. For undergraduates with severe depression, in addition to drug intervention, we analyze it in psychological intervention. Therefore, negative emotions of undergraduates should be corrected in appropriate ways and in time. Finally, the experimental results reveal that the proposed method is superior to baselines with respect to classification accuracy, classification time and recognition rate of negative emotions among undergraduates. This paper uses weight and structure double-determination method to train RBFNN classifier and classify the negative emotions of undergraduates into three categories. Although it effectively reflects the negative emotions of undergraduates, the subdivision is not strong enough. The next step can try to train other types of neural network classifiers to achieve better classification effect.

## Data Availability Statement

The raw data supporting the conclusions of this article will be made available by the authors, without undue reservation.

## Ethics Statement

The studies involving human participants were reviewed and approved by Ethics Committee of Xinxiang University.

## Author Contributions

WZ contributes to the whole paper.

## Conflict of Interest

The author declares that the research was conducted in the absence of any commercial or financial relationships that could be construed as a potential conflict of interest.

## Publisher's Note

All claims expressed in this article are solely those of the authors and do not necessarily represent those of their affiliated organizations, or those of the publisher, the editors and the reviewers. Any product that may be evaluated in this article, or claim that may be made by its manufacturer, is not guaranteed or endorsed by the publisher.
